# Answers to Correspondents

**Published:** 1909-06-26

**Authors:** 


### EDITOR'S LETTER-BOX.

### ANSWERS TO CORRESPONDENTS.

Miss C. F. Hatton is thanked for the reference kindly sent to this office, which has been forwarded to the source of the inquiry.

E. A. C.—The condition mentioned probably has no effect on sexual feeling, and must of necessity be of the nature of a developmental error. Precise information as to the nature of the condition cannot be given from so short a description without seeing the actual parts. It is quite unusual for the clitoris to be divided in the manner described, variations in length and size being the only common malformations. The significance of this condition would be influenced by the condition of the internal sexual organs, the presence or absence of uterus and ovaries, and other sexual characteristics.

Dr. Sinclair.—We believe that Dr. Selig's paper is not published in full in any English medical journal; but we are communicating with the author of our abstract, and will endeavour to supply you shortly with all available information.

